# What Are the Expectations for Cardiac Resynchronization Therapy? A Validation of Two Response Definitions

**DOI:** 10.3390/jcm10030514

**Published:** 2021-02-01

**Authors:** Toshiko Nakai, Yukitoshi Ikeya, Rikitake Kogawa, Naoto Otsuka, Yuji Wakamatsu, Sayaka Kurokawa, Kimie Ohkubo, Koichi Nagashima, Yasuo Okumura

**Affiliations:** Division of Cardiology, Department of Medicine, Nihon University School of Medicine, 30-1 Oyaguchi Kamicho, Itabashi-ku, Tokyo 173-8610, Japan; ikeyayukitoshi@yahoo.ne.jp (Y.I.); rickey1003feb2nd@yahoo.co.jp (R.K.); chibalotte008@gmail.com (N.O.); a.youthful.pine@gmail.com (Y.W.); kurokawa.sayaka@nihon-u.ac.jp (S.K.); kimikimi248@yahoo.co.jp (K.O.); cocakochan@gmail.com (K.N.); okumura.yasuo@nihon-u.ac.jp (Y.O.)

**Keywords:** cardiac resynchronization therapy, responder, NYHA functional class, mortality

## Abstract

Background: The definition of response to cardiac resynchronization therapy (CRT) varies across clinical trials. There are two main definitions, i.e., echocardiographic response and functional response. We assessed which definition was more reasonable. Methods: In this study of 260 patients who had undergone CRT, an echocardiographic response was defined as a reduction in a left ventricular end-systolic volume of greater than or equal to 15% or an improvement in left ventricular ejection fraction of greater than or equal to 5%. A functional response was defined as an improvement of at least one class category in the New York Heart Association functional classification. We assessed the response to CRT at 6 months after device implantation, based on each definition, and investigated the relationship between response and clinical outcomes. Results: The echocardiographic response rate was 74.2%. The functional response rate was 86.9%. Non-responder status, based on both definitions, was associated with higher all-cause mortality. Cardiac death was only associated with functional non-responder status (hazard ratio (HR) 2.65, 95% confidence interval (CI) 1.19–5.46, *p* = 0.0186) and heart failure hospitalization (HR 2.78, 95% CI, 1.29–5.26, *p* = 0.0111). Conclusion: After CRT implantation, the functional response definition of CRT response is associated with a higher response rate and better clinical outcomes than that of the echocardiographic response definition, and therefore it is reasonable to use the functional definition to assess CRT response.

## 1. Introduction

Cardiac resynchronization therapy (CRT) has become the standard treatment for patients with mild to moderate or severe heart failure (HF) [1,2,3,4]. Clinical studies have reported a low CRT response rate of 70%, which may be one explanation for the underutilization of CRT in Japan, as well as all over the world [1,2,3,4,5]. However, we believe that patients in actual clinical practice have a better response than that reported in the literature. There are various definitions of CRT response, and the response rate varies significantly according to the definition [6,7,8]. In general, two definitions have been used in most clinical trials, i.e., echocardiographic response and functional response. An echocardiographic response is defined as a reduction in the left ventricular end-systolic volume (LVESV) of greater than or equal to 15% or an improvement in the left ventricular ejection fraction (LVEF) of greater than or equal to 5%. A functional response is defined as an improvement in the functional status of at least one class category based on the New York Heart Association (NYHA) functional classification, assessed at 6 months after CRT implantation [9]. Generally, an echocardiographic response is used to assess the effect of CRT, but often patients can improve from a status of NYHA class II or III to a NYHA class I status after CRT implantation, even though they are considered to be non-responders based on an echocardiographic assessment. We believe that there is a gap between the echocardiographic and functional response definitions. Thus, in this study, we investigate whether these two commonly used definitions are associated with clinical outcomes and we assess which definition is more reasonable for assessing CRT response.

## 2. Materials and Methods

In this retrospective observational study, we studied 284 consecutive patients who underwent CRT implantation at the Nihon University Hospital between March 2004 and March 2020. The CRT implantation was performed in patients with a QRS duration > 120 milliseconds (ms) and a LVEF ≤ 35%, which correspond to class I, IIa, and IIb indications according to the Japanese Circulation Society guidelines [10]. Twenty-four patients were excluded from the analysis because of loss to follow-up.

### 2.1. Study Design

We classified each patient as a responder or a non-responder using both response definitions based on their clinical functional status and echocardiographic criteria at the 6-month follow-up visit after CRT implantation. Then, we investigated the associations between each type of response and long-term clinical outcomes, including hospitalization due to HF and cardiac death. This study was approved by the Clinical Research Judging Committee of the Nihon University Itabashi Hospital.

### 2.2. Echocardiographic Measurement

End-systolic and end-diastolic left ventricular (LV) volumes were derived from conventional apical 2-chamber and 4-chamber images. LVEF was calculated using the biplane Simpson method [11].

### 2.3. Cardiac Resynchronization Therapy (CRT) Programming

Atrioventricular (AV) delay was optimized using automatic optimization, which was incorporated into each device, in order to achieve a narrower QRS, as compared with the baseline QRS complex. However, if the QRS did not narrow sufficiently with the automatic optimization algorithm, the AV and VV delays were optimized manually while looking at the QRS width on the electrocardiogram. Notably, in patients with atrial fibrillation (AF), the CRT was occasionally reprogrammed at a clinic visit to maintain a higher pacing rate (over 85%).

### 2.4. Statistical Analysis

Continuous variables are presented as means ± SD. Categorical variables are expressed as numbers and percentages. Differences between groups were assessed using Student’s *t*-test for normally distributed continuous variables and the Mann–Whitney U test for non-normally distributed continuous variables. Fisher’s exact test or a chi-squared test was used for categorical variables. Cox regression was used to predict clinical outcomes, including all-cause mortality, HF hospitalization, and cardiac death, in a univariate analysis followed by a multivariate analysis. Survival analysis using the Kaplan–Meier method with the log-rank test was used to analyze cumulative events during the follow-up period. A *p*-value < 0.05 was considered to be significant.

## 3. Results

Patients who underwent CRT implantation, at the 6-month follow-up visit, were classified as responders or non-responders based on the two definitions of response. There were 163 patients classified as echocardiographic responders and 226 patients classified as functional responders. We compared the characteristics of responders and non-responders and investigated the relationships between responder status and the clinical outcomes of all-cause mortality, HF hospitalization, and cardiac death for a mean follow-up duration of 48 ± 45 months.

### 3.1. Patient Characteristics

A total of 260 patients (73% male) with a mean age of 67 years were analyzed. The mean follow-up duration was 48 ± 45 months. One hundred and eighty-six (70%) patients were classified as having NYHA class III. Patients with NYHA class IV had the shortest QRS duration and a higher proportion had ischemic etiology as compared with patients in other groups. Patient characteristics at baseline are shown in Table 1.

### 3.2. CRT Optimization

In sixty-four patients (48%), an automatic optimization algorithm was used and in 196 patients (75%) a manual optimization was performed. The QRS duration was shortened from 152.7 ± 32 to 145.4 ± 25 ms by CRT optimization.

### 3.3. Response Rate

**Figure 1:**
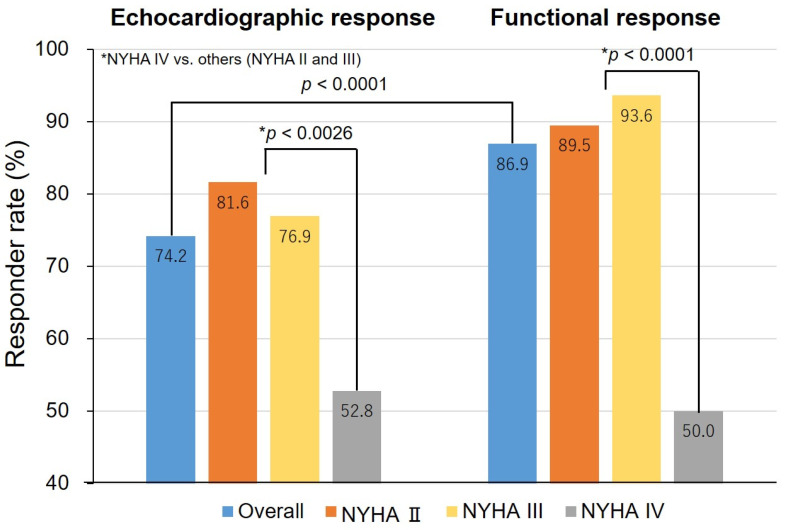
Responder rates, overall and by the New York Heart Association (NYHA) classification. *p*-values were calculated to assess differences among patients with NYHA class IV versus NYHA class II or class III by response criteria. * *p*-value indicates the difference between NHYA IV versus others (NYHA II and III).

The responder rates based on the criteria of both response definitions and the baseline NYHA functional class are shown in Figure 1. The proportion of echocardiographic and functional responders was 74.2% and 86.9%, respectively (*p* < 0.0001). Among the patients with mild to moderate HF, the response rate was higher for functional responders than echocardiographic responders. However, the response rate in patients with NYHA functional class IV was only about 50% for both types of responders, which was significantly lower than that in patients with NYHA functional class II or III.

The characteristics of responders and non-responders for each response definition are shown in Table 2. Echocardiographic non-responders included more patients with severe HF (NYHA class IV) (*p* = 0.0026) and ischemic etiology as compared with echocardiographic responders (*p* = 0.0266). Similar results were seen with functional responders and non-responders. However, the NYHA class IV classification seemed to have the most influence on the response to CRT.

Response discrepancy between two criteria was detected in 39 (15%) patients (Table 3). There was a higher percentage of chronic obstructive pulmonary disease (COPD) in patients with response discrepancy (*p* = 0.0409). Other variables were not significantly different between the patients with and without response discrepancy. Considering the breakdown of response discrepancy type, thirty-three patients (85%) were classified as functional responders and echocardiographic non-responders, and six patients (15%) were classified as echocardiographic responders and functional non-responders. There was a significant difference in the percentage of severe HF patients (NYHA IV) with response discrepancy between the two definitions of response.

### 3.4. Predictors of Nonresponse to CRT

Predictors of echocardiographic nonresponse included NYHA class IV, ischemic etiology, and the absence of beta-blocker therapy. Predictors of functional nonresponse included non-left bundle branch block (NLBBB) morphology, NYHA class IV, and ischemic etiology. Multivariate analysis revealed that NYHA class IV was the strongest predictor of nonresponse for both echocardiographic and functional criteria (Table 4).

### 3.5. Hospitalization Due to Heart Failure (HF)

**Figure 2:**
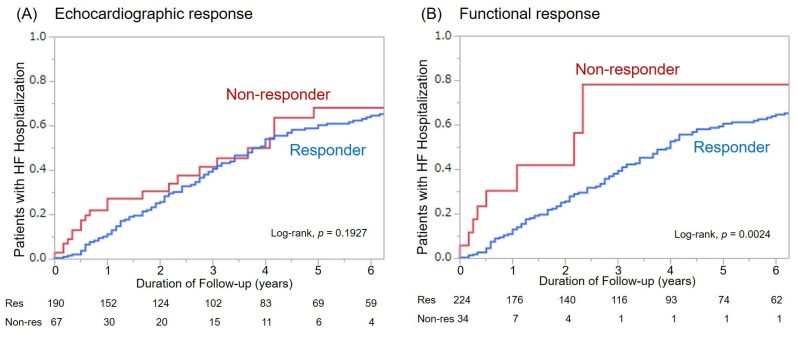
Hospitalization for heart failure among responders and non-responders based on both response definitions. (**A**): Echocardiographic response, (**B**): Functional response.

Among 260 patients, 90 patients (35%) were hospitalized during follow-up. The Kaplan–Meier analysis showed that the cumulative number of patients with HF hospitalization was not significantly different between echocardiographic responders and non-responders (*p* = 0.1927). By contrast, there was a significant difference in the cumulative number of patients with HF hospitalization between functional responders and non-responders (*p* = 0.0024) (Figure 2)

### 3.6. Mortality

**Figure 3:**
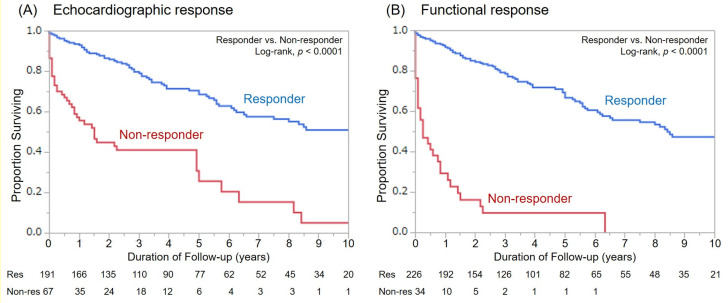
Kaplan–Meier curves for all-cause mortality among responders and non-responders based on both response definitions. (**A**): Echocardiographic response, (**B**): Functional response.

**Figure 4:**
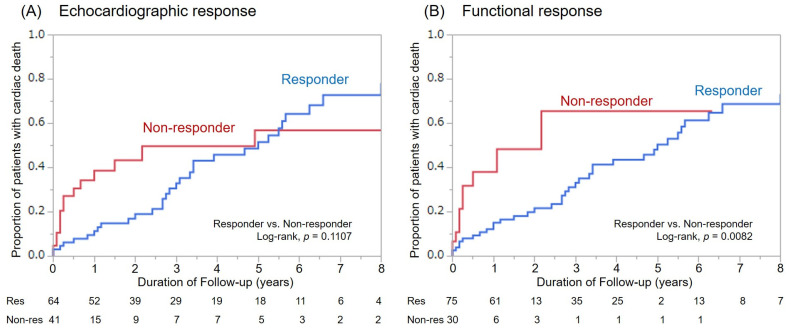
Cumulative number of cardiac deaths among responders and non-responders based on both re-sponse definitions. (**A**): Echocardiographic response, (**B**): Functional response.

For the mean follow-up duration of 48 ± 45 months, there were 109 patient deaths. Fifty-six deaths were classified as cardiac deaths, among whom 11 died suddenly from VT/VF (SCD), and all other patients died of heart failure. Overall survival was better in responders as compared with non-responders for both response definitions (Figure 3). However, there was only a significant difference in the cumulative number of cardiac deaths between functional responders and non-responders (*p* = 0.0082, Figure 4).

### 3.7. Relationship between non-Responder Status and Clinical Outcomes

Cox regression analysis confirmed a stronger association with non-responder status and clinical outcomes for functional versus echocardiographic responders (Table 5). All-cause mortality was associated with both echocardiographic and functional response (*p* = 0.0334 and *p* < 0.0001, respectively). However, echocardiographic non-responder status was not associated with cardiac death (*p* = 0.1257) or HF hospitalization (*p* = 0.2115). Only functional non-responder status was significantly associated with cardiac death (*p* = 0.0186) and HF hospitalization (*p* = 0.0111).

## 4. Discussion

In this study, functional responders were associated with better clinical outcomes after CRT implantation, especially with regard to HF hospitalization and cardiac death. A functional response was more strongly associated with clinical outcomes than an echocardiographic response.

The definition of CRT response has varied in previous clinical trials [6,7,8]. However, the most common definition of CRT response includes echocardiographic parameters describing reverse remodeling [12,13,14], perhaps because echocardiographic data are more objective than symptoms, and some patients feel better after treatment and may experience placebo effects. Thus, many clinical trials use the NYHA functional class, as well as echocardiographic parameters, to define the response to CRT. Our study confirmed that the functional response definition is associated with subjective symptoms and could also be associated with better outcomes following CRT implantation.

The CRT response rate has remained at around 70% in the decade since the first trial of the CRT MIRACLE (Multicenter InSync Randomized Clinical Evaluation) study [1,2,3,4,5,15,16]. Our findings regarding echocardiographic response are in agreement with findings from previous studies. There was a higher proportion of echocardiographic non-responders who had ischemic cardiomyopathy (ICM). Reverse remodeling is relatively more limited in ICM, as compared with other etiologies associated with large scar areas [17,18]. From the MADIT CRT (Multicenter Automatic Defibrillator Implantation Trial with Cardiac Resynchronization Therapy) trial, Barsheshet et al. also reported a difference in the proportion of patients with echocardiographic response, based on whether they had ICM. They concluded that the risk assessment for a CRT defibrillator should be etiology specific [19]. In previous trials, more than half of the patients had ischemic etiology, and the response rate was 70%, which was based mostly on echocardiographic criteria [1,2,3,15]. However, even in patients classified as echocardiographic non-responders, intraventricular conduction disturbance was definitely corrected, as indicated by a narrower QRS after CRT. Through this correction of conduction disturbance, HF can improve, to some extent. In fact, in our study, patients without reverse remodeling had improvements in symptom status and lived longer.

The CAVIAR (CRT age and vectorcardiographic QRS area-interventricular mechanical delay and apical rocking) score was proposed by Maass et al. as an echocardiographic marker for CRT response [20]. They demonstrated the association of the CAVIAR score with the amount of reverse remodeling, as well as the clinical outcomes. Their findings were in agreement with previous studies reporting that reverse remodeling was associated with better clinical outcomes. However, reverse remodeling depends on the etiology [17,18,19,21]. Martens et al. reported that the cut-off in the LVEF for predicting a good clinical outcome was 5.5% in ICM vs. 10.5% in non-ICM [22]. They also confirmed that patients with ICM derive benefits, although there is a slight degree of reverse remodeling. On the basis of those data, CRT response should not be evaluated using only echocardiographic parameters but should also use clinical outcomes. Quantifiable numerical values, such as echocardiographic parameters, are a convenient form of evaluation and, therefore, have been used as the standard response criteria. However, the aim of CRT is to correct cardiac conduction disorders using a pacing system and, therefore, improve patients’ quality of life. Reverse remodeling is not essential, it is the result of CRT.

The ADVANCE CRT (Advance Cardiac Resynchronization Therapy) registry revealed that a site-specific assessment underestimated non-responders as compared with an objective measure of clinical composite score (CCS), thus overlooking higher-risk individuals [23]. They also mentioned the underutilization of remote monitoring and fewer additional treatments for non-responders. They emphasized that it is important to make an accurate diagnosis of a non-responder (using CCS) and actively manage the patient’s treatment, including medication, device reprogramming, or other interventions if needed.

Mullens et al. published a position statement on recommendations for managing patients who have undergone CRT, and patients who are future candidates for CRT [24]. They addressed the underutilization of CRT, the definition of CRT response, and the optimization of post-implant CRT care. They highlighted the issue of patients with HF who are managed in non-specialist care, which may result in delaying CRT. The situation is similar in Japan. It is difficult to have specialists at all institutes or hospitals; however, better communication between specialists and primary physicians should improve the understanding and education of CRT.

In our study, we assessed the functional response using only the NYHA functional classification; thus, this assessment may have also underestimated the non-responders. However, we assessed the response at the 6-month visit, and also each time a patient visited the clinic for medication adjustments and for device reprogramming if needed to achieve an effective CRT. In fact, it may be better to assess CRT response at 6 months after CRT implantation, because patients are often continuously managed up to 6 months after CRT implantation. Rickard et al. reported the survival effect of CRT LVEF improvement, i.e., an “echocardiographic response” [25]. They studied 526 patients and, among those patients, 196 (37.3%) were classified as non-responders. In our study, 67 of 260 patients (25.8%) were echocardiographic non-responders, which was relatively low; possible reasons for this are the smaller number of patients with ischemic etiology (68% versus 35%), AF (50% versus 22%), COPD (20% versus 2%), and the larger number of patients with LBBB morphology (66% versus 30%). However, the long-term survival of more than 5 years was quite similar in both responders and non-responders, based on the echocardiographic response definition. The prognosis may be influenced by a patient’s background and the presence of comorbidities may be difficult to predict. We continue the management of patients with the belief that short-term responses will lead to long-term effects.

Our data show that CRT is a very effective treatment, indeed, far more effective than is commonly thought. The understanding of a 70% response rate is mostly based on the echocardiographic response definition in studies with many patients who had ICM. We emphasize that the objectives of CRT are to improve the quality of life and prolong life and, therefore, based on these objectives, an echocardiographic response definition based on reverse remodeling is not essential. We only hope that these patients remain in good health and happy until the end of their lives.

### 4.1. Study Limitations

First, this study is a single-center retrospective observational study, with a small number of patients. The distributions of HF etiology or gender may be different at other institutions or in other countries [1,2,3,15]. However, Japan is known to have a relatively lower percentage of patients with ischemic etiology [26,27] than Western countries. Considering that reverse remodeling is more limited in ICM, the present results may underestimate the echocardiographic response rate due to the small percentage of study patients with ischemic etiology. However, there was a significant difference in the relationship between responder status and better clinical outcomes for both criteria, although the study included only a small number of patients with ischemic etiology.

### 4.2. Clinical Implications

The nonresponse rate for CRT is a significant concern, which discourages the use of this treatment. However, depending on the definition of response, there might not be as many non-responders as expected. In addition, considering that functional responders can correctly predict better prognoses, we encourage using CRT in as many patients with HF, as is possible and appropriate, according to current guidelines.

## 5. Conclusions

We investigated two definitions of response to CRT, i.e., echocardiographic response and functional response. The CRT response rate differs by response criteria. The functional response is significantly associated with better outcomes. If the purpose of CRT is to improve quality of life or prolong life, it may be sufficient to use functional criteria to assess response.

## Figures and Tables

**Table 1:** Baseline characteristics and medication.

	Overall (*n* = 260)	NYHA II (*n* = 38)	NYHA III (*n* = 186)	NYHA IV (*n* = 36)	*p*-Value (NYHA IV vs. Others)
**Age (years), mean**	67.3	69.2	67.5	64.3	0.1076
**Males, *n* (%)**	204 (78)	31 (82)	142 (76)	31 (86)	0.2290
**QRS duration (ms), mean**	152.7	156.9	153.9	141.8	0.0233
**LBBB morphology, *n* (%)**	172 (66)	23 (68)	123 (66)	23 (64)	0.7580
**LVEF (%), mean**	29.1	31.2	29.1	26.9	0.1998
**Ischemic etiology, *n* (%)**	91 (35)	15 (39)	57 (31)	19 (53)	0.0160
**Atrial fibrillation, *n* (%)**	58 (22)	11 (29)	41 (22)	6 (17)	0.3101
**Hypertension, *n* (%)**	130 (50)	25 (66)	93 (50)	12 (33)	0.0298
**Diabetes mellitus, *n* (%)**	98 (38)	17 (45)	64 (34)	17 (47)	0.2086
**COPD, *n* (%)**	6 (2)	0 (0)	5 (3)	1 (3)	0.8437
**eGFR (mL/min/1.73 m^2^), mean**	51.0	46.9	52.1	49.3	0.6354
**CRT pacemaker, *n* (%)**	96 (37)	14 (37)	70 (38)	12 (33)	0.6285
**Upgrade from pacemaker, *n* (%)**	52 (20)	10 (26)	38 (20)	4 (11)	0.1276
**Medication**					
**Beta blocker, *n* (%)**	230 (91)	34 (92)	167 (92)	29 (85)	0.2209
**ACEI or ARB, *n* (%)**	169 (67)	28 (76)	123 (68)	18 (53)	0.0652
**Diuretic, *n* (%)**	225 (89)	34 (92)	158 (87)	33 (89)	0.1045

NYHA, New York Heart Association class; LBBB, left bundle branch block; LVEF, left ventricular ejection fraction; COPD, chronic obstructive pulmonary disease; ACEI, angiotensin-converting enzyme inhibitor; ARB, angiotensin receptor blocker.

**Table 2:** Characteristics of responders and non-responders.

	Echocardiographic			Functional		
	Responder (*n* = 193)	Non-Responder (*n* = 67)	*p*-Value	Responder (*n* = 226)	Non-Responder (*n* = 34)	*p*-Value
**Age (years), mean**	67.4	67.0	0.8182	67.5	66.4	0.6411
**Males, *n* (%)**	147 (76)	57 (85)	0.1156	175 (77)	29 (85)	0.2801
**QRS duration (ms), mean**	154.1	148.6	0.2065	154.0	144.0	0.0794
**LBBB morphology, *n* (%)**	136 (71)	44 (66)	0.4667	160 (71)	20 (59)	0.1676
**NYHA IV, *n* (%)**	19 (10)	17 (25)	0.0026	18 (8)	18 (53)	<0.0001
**LVEF (%), mean**	28.9	29.7	0.6460	29.6	26.3	0.1150
**Ischemic etiology, *n* (%)**	60 (31)	31 (46)	0.0266	74 (33)	17 (50)	0.0492
**Atrial fibrillation, *n* (%)**	39 (20)	19 (28)	0.1751	48 (21)	10 (29)	0.2993
**Hypertension, *n* (%)**	99 (51)	31 (46)	0.4782	114 (50)	16 (47)	0.7129
**Diabetes mellitus, *n* (%)**	71 (37)	27 (40)	0.6104	82 (36)	16 (47)	0.2318
**COPD, *n* (%)**	3 (2)	3 (4)	0.1990	6 (3)	0 (0)	0.1919
**eGFR (mL/min/1.73 m^2^), mean**	51.1	50.6	0.8808	51.8	45.8	0.1434
**CRT-Pacemaker, *n* (%)**	75 (39)	21 (31)	0.2682	86 (38)	10 (29)	0.3234
**Upgrade from pacemaker, *n* (%)**	44 (23)	8 (12)	0.0458	48 (21)	4 (12)	0.1741
**Medication**						
**Beta** **Blocker, *n* (%)**	179 (93)	59 (89)	0.4017	212 (94)	26 (79)	0.0098
**ACEI or ARB, *n* (%)**	136 (70)	37 (56)	0.0343	155 (69)	18 (55)	0.1171
**Diuretic, *n* (%)**	172 (89)	59 (89)	0.9504	201 (89)	30 (91)	0.7279

NYHA, New York Heart Association class; LBBB, left bundle branch block; LVEF, left ventricular ejection fraction; COPD, chronic obstructive pulmonary disease; ACEI, angiotensin-converting enzyme inhibitor; ARB, angiotensin receptor blocker.

**Table 3:** Characteristics of patients with response discrepancy between two criteria.

				Breakdown of Response Discrepancy (*n* = 39)		
	Response Discrepancy (*n* = 39)	No Response Discrepancy (*n* = 221)	*p*-Value	Functional Response without Echocardiographic Response (*n* = 33)	Echocardiographic Response without Functional Response (*n* = 6)	*p*-Value
**Age (years), mean**	67.8	67.0	0.7127	68.5	64.2	0.3814
**Males, *n* (%)**	33 (85)	169 (76)	0.2430	28 (85)	5 (83)	0.9253
**QRS duration (ms), mean**	149.1	153.3	0.4464	150.7	140.3	0.3042
**NYHA IV, *n* (%)**	5 (13)	31 (14)	0.8392	2 (6)	3 (50)	0.0110
**LBBB morphology, *n* (%)**	30 (73)	150 (68)	0.5473	22 (67)	3 (50)	0.4414
**LVEF (%), mean**	30.1	29.7	0.8482	31.0	24.2	0.1629
**Ischemic etiology, *n* (%)**	18 (46)	73 (33)	0.1189	15 (45)	3 (50)	0.8374
**Atrial fibrillation, *n* (%)**	12 (31)	48 (22)	0.2289	10 (30)	2 (33)	0.8831
**Hypertension, *n* (%)**	21 (54)	109 (49)	0.6022	16 (48)	5 (83)	0.0997
**Diabetes mellitus, *n* (%)**	12 (31)	86 (39)	0.3275	10 (30)	2 (33)	0.8831
**COPD, *n* (%)**	3 (8)	3 (1)	0.0409	3 (9)	0 (0)	0.3062
**eGFR, (ml/min/1.73 m^2^), mean**	51.1	48.4	0.5596	52.8	39.2	0.2372
**CRT-Pacemaker, *n* (%)**	15 (38)	81 (37)	0.8294	13 (39)	2 (33)	0.7773
**Upgrade from pacemaker, *n* (%)**	7 (18)	45 (20)	0.7254	5 (15)	2 (33)	0.3178
**Beta blocker, *n* (%)**	37 (95)	201 (91)	0.4348	32 (97)	5 (83)	0.2354
**ACEI or ARB, *n* (%)**	25 (64)	148 (67)	0.6999	20 (60)	5 (83)	0.2612
**Diuretic, *n* (%)**	35 (90)	196 (89)	0.9031	29 (88)	6 (100)	0.2339

NYHA, New York Heart Association class; LBBB, left bundle branch block; LVEF, left ventricular ejection fraction; COPD, chronic obstructive pulmonary disease; ACEI, angiotensin-converting enzyme inhibitor; ARB, angiotensin receptor blocker.

**Table 4:** Predictors of nonresponse by response criteria.

	Echocardiographic				Functional			
	Univariate OR (95% CI)	*p*-Value	Multivariate OR (95% CI)	*p*-Value	Univariate OR (95% CI)	*p*-Value	Multivariate OR (95% CI)	*p*-Value
**Age**	0.10 (0.98–1.02)	0.8178			0.99 (0.96–1.02)	0.6429		
**Male Gender**	1.69 (0.67–5.16)	0.2801			1.78 (0.87–3.96)	0.1156		
**QRS duration**	0.99 (0.98–1.00)	0.0729			0.99 (0.98–1.00)	0.2017		
**LBBB morphology**	0.68 (0.39–1.22)	0.1964			0.40 (0.19–0.82)	0.0136	0.33 (0.14–0.76)	0.0097
**NYHA IV**	3.11 (1.51–6.44)	0.0022	2.76 (1.31–5.82)	0.0076	13.0 (5.68–29.8)	<0.0001	13.5 (5.64–32.5)	<0.0001
**LVEF**	0.96 (0.92–1.01)	0.1028			1.01 (0.98–1.04)	0.6452		
**Ischemic etiology**	2.05 (0.99–4.28)	0.0538	1.70 (0.94–5.82)	0.0793	1.91 (1.08–3.38)	0.0266	1.98 (0.87–4.52)	0.1032
**Atrial fibrillation**	1.55 (0.67–3.37)	0.2993			1.56 (0.82–2.93)	0.1751		
**Hypertension**	0.82 (0.47–1.43)	0.4782			0.87 (0.42–1.80)	0.7131		
**Diabetes mellitus**	1.16 (0.66–2.05)	0.6104			1.56 (0.76–3.23)	0.2293		
**COPD**	2.97 (0.58–15.1)	0.1990			0.01 (0.01–0.21)	0.9915		
**eGFR**	0.10 (0.99-1.01)	0.8802			0.99 (1.00–7.18)	0.1354		
**CRT-Pacemaker**	0.72 (0.40–1.30)	0.2682			0.68 (0.31–1.49)	0.3325		
**Upgrade from pacemaker**	0.46 (0.20–1.03)	0.0458	0.57 (0.25–1.31)	0.1819	0.49 (0.17–1.47)	0.2057		
**Beta blocker**	0.21 (0.08–1.56)	0.0028	0.74 (0.27–2.05)	0.5636	0.47 (0.19–1.17)	0.1026		
**ACEI or ARB**	0.56 (0.26–1.21)	0.1391			0.66 (0.37–1.20)	0.1750		
**Diuretic**	1.18 (0.38–5.20)	0.7889			1.21 (0.50–3.43)	0.6842		

NYHA, New York Heart Association class; LBBB, left bundle branch block; LVEF, left ventricular ejection fraction; COPD, chronic obstructive pulmonary disease; ACEI, angiotensin-converting enzyme inhibitor; ARB, angiotensin receptor blocker; OR, odds ratio; CI, confidence interval.

**Table 5:** Cox regression analysis of all-cause mortality, cardiac death, and hospitalization for both echocardiographic and functional responders.

	Mortality		Cardiac Death		HF Hospitalization	
	HR (95% CI)	*p*-Value	HR (95% CI)	*p*-Value	HR (95% CI)	*p*-Value
**Echocardiographic Non-responder**	1.86 (1.05–3.15)	0.0334	1.61 (0.34–1.15)	0.1257	1.32 (0.85–1.99)	0.2115
**Functional Non-responder**	6.45 (3.54–12.0)	<0.0001	2.65 (1.19–5.46)	0.0186	2.78 (1.29–5.26)	0.0111

HF, heart failure; HR, hazard ratio; CI, confidence interval.

## Data Availability

The data in this study are available on request from the corre-sponding author.
